# Modulation of Mouse Embryonic Stem Cell Proliferation and Neural Differentiation by the P2X7 Receptor

**DOI:** 10.1371/journal.pone.0096281

**Published:** 2014-05-05

**Authors:** Talita Glaser, Sophia La Banca de Oliveira, Arquimedes Cheffer, Renata Beco, Patrícia Martins, Maynara Fornazari, Claudiana Lameu, Helio Miranda Costa Junior, Robson Coutinho-Silva, Henning Ulrich

**Affiliations:** 1 Departamento de Bioquímica; Instituto de Química, Universidade de São Paulo, São Paulo, Brasil; 2 Instituto de Biofísica Carlos Chagas Filho, Universidade Federal do Rio de Janeiro - UFRJ, Rio de Janeiro, RJ, Brazil; University of Freiburg, Germany

## Abstract

**Background:**

Novel developmental functions have been attributed to the P2X7 receptor (P2X7R) including proliferation stimulation and neural differentiation. Mouse embryonic stem cells (ESC), induced with retinoic acid to neural differentiation, closely assemble processes occurring during neuroectodermal development of the early embryo.

**Principal Findings:**

P2X7R expression together with the pluripotency marker Oct-4 was highest in undifferentiated ESC. In undifferentiated cells, the P2X7R agonist Bz-ATP accelerated cell cycle entry, which was blocked by the specific P2X7R inhibitor KN-62. ESC induced to neural differentiation with retinoic acid, reduced Oct-4 and P2X7R expression. P2X7R receptor-promoted intracellular calcium fluxes were obtained at lower Bz-ATP ligand concentrations in undifferentiated and in neural-differentiated cells compared to other studies. The presence of KN-62 led to increased number of cells expressing SSEA-1, Dcx and β3-tubulin, as well as the number of SSEA-1 and β3-tubulin-double-positive cells confirming that onset of neuroectodermal differentiation and neuronal fate determination depends on suppression of P2X7R activity. Moreover, an increase in the number of Ki-67 positive cells in conditions of P2X7R inhibition indicates rescue of progenitors into the cell cycle, augmenting the number of neuroblasts and consequently neurogenesis.

**Conclusions:**

In embryonic cells, P2X7R expression and activity is upregulated, maintaining proliferation, while upon induction to neural differentiation P2X7 receptor expression and activity needs to be suppressed.

## Introduction

Purinergic receptors are classified as P1 adenosine and P2 ATP receptors based on their selectivity for adenosine and nucleotide agonists. While P1 and P2Y subtypes are G-protein-coupled metabotropic receptors, P2X receptors are resembled as homo- or hetero-trimeric ligand-gated ion channels from seven possible subunits. The ion channels formed by P2X1-P2X7 subunits are permeable to Na^+^, K^+^ and Ca^2+^ ions, while at high agonist concentrations P2X7 receptor (P2X7R) subtypes assemble cation ion channels that are capable of pore forming, allowing the unselective flow of compounds with molecular masses of 700Da besides the uncontrolled entry of ions, including Ca^2+^, into the cell which may induce intrinsic cell death programs [1], [2], [3]. Moreover, the P2X7R has an intracellular domain that couples receptor activation to intracellular signaling events and is classically involved with apoptosis [4], [5]. However, P2X7 receptors have also been involved in cell survival and increased proliferation of cancer cells [4], [6], [7], [8] at low extracellular ATP concentration [9]. These divergent roles can be explained by the fact that now is possible to dissociate the channel from pore function, and therefore these might be two separate molecular entities [10], [11].

Being expressed in almost every cell and attributed to multiple cellular functions, purinergic receptors have been detected in early embryonic development [12]. P2 receptor antagonists injected into the early gastrula (first invagination) stage of the *Xenopus* embryo, impaired development with embryos having no head, trunk, somite and notochord and sometimes no tail; in midway gastrula, the embryos had no heads, but with trunks and tails [13].

Maiken Nedergaard's group showed that neuronal differentiation is accompanied by a marked down-regulation of purinergic signaling and the neural progenitor cells themselves were the source of local ATP secretion [14]. Furthermore in the brain of newborn rats a 6 kb RNA was detected corresponding to the P2X7R transcript, which was not detectable in adult brains [15], suggesting possible developmental functions of the P2X7R.

Complex developmental mechanisms are often studied in simplified environment by using stem cell models. Embryonic stem cells (ESC) are isolated from blastocysts inner cell mass maintaining *in vitro* their capability of self-renewal, proliferating in an undifferentiated state, being pluripotent (capable to differentiate into all cell types of an adult organism) and having a stable karyotype [5], [16]. Besides their contribution to elucidation of developmental mechanisms, ESC have been extensively studied during last decades as a promise to cure diverse diseases and injuries. In this study we used E14TG2a cell line, because beyond maintaining ESC characteristics, these cells can grow in feeder free cultures, avoiding contamination by fibroblasts during differentiation process [17], [18].

Extracellular ATP induces proliferation and regulates proliferation in pluripotent stem cell models expressing various purinergic receptor subtypes [5], [19], [20], [21], [22], [23]. Here, we provide evidence for so far unknown roles of the P2X7R in embryonic stem cell biology including maintenance of proliferation and induction to neuroectodermal differentiation.

## Methods

### P2X7R (−/−) knock-out mice

P2X7 (−,−) knock-out mice, developed by the method of Dr James Mobley (PGRD, Pfizer Inc, Groton, CT, USA) were housed in controlled temperature of 22±2°C and 60-70% humidity and unlimited access to food and water *ad libitum* under 12 h light-dark cycle. Animal maintenance and sacrificing for isolation of whole-brain tissue was in agreement with the regulations of the Local Animal Ethics Committee of the Federal University of Rio de Janeiro, Brazil.

### Culture and differentiation of E14Tg2A mouse embryonic stem cell

The feeder cell independent E14Tg2A embryonic stem (ES) cell line was kindly provided by Dr. Deborah Schechtman, Instituto de Química, University of São Paulo. Cells were first isolated by Hooper et al, 1987 [18] and further characterized by Magin et al. 1992 [18]. Cells were cultured as described by Fornazari and coworkers [24]. Basically, cells were grown in DMEM containing 15% Fetal Bovine Serum (FBS), 2 mM sodium pyruvate, 1% non-essential amino acids, 10^3^ U/mL Leukemia Inhibitory Factor (LIF), 0.1 mM β-mercaptoethanol and 10 mM HEPES, pH 7.4, at 37°C in a water-saturated atmosphere containing 5% CO2. For neural differentiation, 5X10^6^ cells were cultured in 90×15 mm non-adherent plates in DMEM supplemented with 20% FBS, 1% non-essential amino acids and 0.1 mM β-mercaptoethanol for 48 h to induce embryoid body formation. Following substitution of the culture medium, cells were cultured as a suspension for 4 further days in the presence of 5 µM retinoic acid. Embryoid bodies were seeded in 125 mm adherent cell culture flasks and grown for further 4 or 12 days.

### ESC growth curve assay

Cells were plated at a 30×10^4^ density in p35 mm previous gelatinized plates. Cells were removed from duplicate plates by use of trypsin and resuspended in 1 ml of Hanks' balanced salt solution containing 0.5% formalin to fix them after 8, 12, 24, 36, 48 and 60 h. Cell numbers were determined with a Neubauer chamber (0.100 mm depth, 0.0025 mm^2^ area) on an inverted microscope Axiovert 200 (Zeiss).

### Protein extraction and western blotting assays

For preparation of cell lysates, E14Tg2A undifferentiated and differentiated cells were trypsinized, centrifuged for 10 min at 400 g, washed with PBS and centrifuged again. The pellet was then dissolved in lysis buffer (20 mM Tris-HCl, 1 mM EDTA, 0.5% NP40, 20% Glycerol ph = 7,5) plus a protease inhibitor cocktail (Thermo Life Sciences) and phosphatase inhibitors (2 mM orthovanadate and 5 mM sodium fluoride, Thermo Life Sciences), incubated for 15 min on ice, and then centrifuged for 25 min at 2000×g and 4°C. The same procedure was performed with total brain lysates of P2X7R (−/−) animals.

Protein quantification was measured by the Coomassie Blue method [33] with bovine serum albumin as the standard. Thirty micrograms of protein in sample buffer were separated by SDS-PAGE on a 10% polyacrylamide gel at a constant voltage of 140 V. Then proteins were transferred onto a nitrocellulose membrane (Thermo-scientific) in a wet system for 1 hour at constant amperage of 400 mA. For blocking of nonspecific binding, 5% BSA in TBS-T was added for 30 min under agitation at room temperature. The membranes were then incubated with primary antibodies for Oct-4 (polyclonal rabbit 1∶1000 Millipore), P2X7 receptor extracellular epitope (monoclonal rabbit 1∶2000 AbCam), P2X7 receptor C-terminus epitope (polyclonal rabbit 1∶1000 Alomone) and α-actin (1∶1000 Sigma-Aldrich) overnight at 4°C. Membranes were then washed and probed with the respective secondary antibodies, Alexa Fluor 488 or 647 (Invitrogen, Life Technologies), for 1 h under agitation at room temperature. Primary and secondary antibodies were diluted in 1% BSA and TBS-T. Membranes were washed in TBS-T and scanned with Typhoon– GE Healthcare. The resulting bands were subjected to densitometric analysis with the ImageJ software. Oct-4 and P2X7R levels were normalized by comparison to β-actin expression.

### Calcium-imaging in E14Tg2A embryonic stem single cells

Undifferentiated and differentiated *E14Tg2A ESC* were loaded with 5 µM of Fluo3-AM for 45 min at 37°C in DMEM High glucose in 0.5% Me2SO and 0.06% of the nonionic surfactant pluronic acid F-127 (Sigma Aldrich). After loading with Fluo-3AM, the cells were incubated with extracellular buffer (140 mM NaCl, 3 mM KCl, 1 mM MgCl_2_, 2 mM CaCl_2_, 10 mM HEPES, 10 mM glucose at pH 7.4) [25]. Ca^2+^ imaging was performed by using the Inverted Research Microscope ECLIPSE-TiS (Nikon, Melville, NY) equipped with a 14 bit high-resolution CCD camera CoolSNAP HQ2 (Photometrics, Tucson, AZ) and analyzed with NIS-Element software (Nikon) using image acquisition rates of two frames per second. Fluo-3 fluorescence was excited with a xenon lamp at 488 nm, and the emitted light was detected using a band-pass filter at 515–530 nm. Intracellular calcium influx was monitored in cells stimulated with 10 µM ATP and 10 µM Bz-ATP. Forty cells were analyzed for each data point and the calcium influx was determined as mean variation between Fluo-3AM fluorescence intensities obtained during the stimulus (F) and the rest state (F_o_), normalized by its basal fluorescence (F_b_). (F-F_o_)/F_b_.

### Calcium measurements by microfluorimetry

Changes in [Ca^2+^]_i_ were determined by microfluorimetry using the FlexStation III (Molecular Devices Corp., Sunny Valley, CA), following the instructions of the manufacturer [26]. Briefly, for undifferentiated cells, they were seeded a night before starting the experiment at a density of 5–3×10^4^ cells/well and for 8 days differentiated cells, 2 EBs/well were seeded in 96-well black microplate with clear bottom, with 100 µl of cell culture medium per well. Cells were incubated for 60 min at 37°C with the FlexStation Calcium Assay Kit (Molecular Devices Corp.) containing 2.5 mM probenecid in a final volume of 200 µl per well. Fluorescence of samples was excited at 485 nm, and fluorescence emission was detected at 525 nm.

Samples were read at 1.52 s intervals for 120 s with a total of 79 read-outs per well. Following 20 s of monitoring basal fluorescence intensity for [Ca^2+^]_i_ levels of nonstimulated cells, agonists (ATP and Bz-ATP) were applied onto the cells, and induced-[Ca^2+^]_i_ transients were monitored for up to 200 s. Responses to agonist addition were determined as peak fluorescence minus the basal fluorescence intensity using the SoftMax2Pro software (Molecular Devices Corp.). Data were expressed as mean values ± standard errors (S.E.).

### Immunofluorescence staining assay

For immunofluorescence detection of specific marker proteins for respective differentiation stages, E14Tg2A cells were grown and induced to differentiate on rounded coverslips (1 cm diameter). Cells were fixed with 4% paraformaldehyde in phosphate-buffered saline (PBS) for 15 min, washed three times with PBS and then incubated for 30 min in a blocking solution containing 0.05% Triton-X 100 and 2% FBS. Cells were incubated overnight at 4°C with primary antibodies raised against mouse monoclonal anti stage-specific embryonic antigen-1 (SSEA-1) (1∶200, Chemicon, Bioscience Research Reagents, Temecula, CA), rabbit polyclonal anti-βIII tubulin (1∶200, Millipore, Billerica, MA), Oct-4 rabbit polyclonal (1∶1000 Millipore), rabbit monoclonal P2X7R (1∶2000 AbCam, Cambridge, MA) and nestin (1∶1000 Millipore) antibodies. The slides were washed three times with PBS, followed by one hour incubation with Alexa Fluor 488 or 555 goat anti-mouse (1∶800, Sigma). In control experiments, the primary antibody was omitted, and immunostaining was never observed. Counterstaining of cell nuclei was achieved with 0.1% of 4′,6-diamidino-2-phenylindole (DAPI). After washing with PBS, the slides were mounted with Vectashield (Vector Laboratories, Burlingame, CA) and examined on an Axiovert 200 epifluorescence microscope (Zeiss, Aalen, Germany), equipped with a Nikon DMX1200F camera and Metamorph image analysis program or on a confocal microscope (Zeiss LSM 780-NLO Multiphoton) and analyzed with the LSMib software (Zeiss).

### Real time polymerase chain reaction

Total RNA was extracted from undifferentiated and ESC subjected to 8 days of neural differentiation using the TRIzol Reagent (Invitrogen) following manufacturer's instruction. All samples were further treated with amplification grade DNase I (Sigma-Aldrich). Reverse transcription for cDNA synthesis was carried out on a thermal cycler using the RevertAid Reverse Transcriptase (Thermo Scientific Fermentas) first strand synthesis system according to the manufacturer's protocol (Invitrogen) in the presence of specific primers listed in (Table 1). The transcription rates of selected mRNAs were measured by real time PCR using the ABI Step One Plus instrument (Life Technologies). Real time PCR was performed in 15 µl of buffer reaction containing of 1 ug cDNA, SYBR Green Master Mix (Life Technologies), and 5 pmol of each sequence-specific primers (Table 1). Thermal cycling conditions consisted of a denaturation for 10 min at 95°C followed by 40 cycles for denaturation for 15 s at 95°C, and annealing/extension for 1 min at 60°C, followed by melting curve analysis. The comparative 2^−ΔΔCT^ method was employed for relative quantification of gene expression as described previously [27] using glyceraldehyde-3-phosphate dehydrogenase (GAPDH) gene expression as an internal standard for normalization.

**Table 1:** Primer sequences for PCR experiments.

Gene	Forward (5′-3′)	Reverse (5′-3′)
**GAPDH**	TGCACCACCAACTGCTTAG	GGATGCAGGGATGATGTTC
**SSEA-1**	CGGACCGACTCGGATGTCT	TTGGATCGCTCCTGGAATAGA
**Dcx**	GAGTGGGGCTTTCGAGTGAT	AAAGAAAGCCGTGTGCCTTG
**β3-tubulin**	AGA CCT ACT GCA TCG ACA ATG AAG	GCT CAT GGT AGC AGA CAC AAG G
**Nestin**	GAG AGT CGC TTA GAG GTG CA	CCA CTT CCA GAC TCC GGG AC
**Oct-4**	ATG CCG TGA AGT TGG AGA AG	TGT ACC CCA AGG TGA TCC TC
**P2X7R**	GCACGAATTATGGCACCGTC	CCCCACCCTCTGTGACATTCT
**IsoA**	TGAGACAAACAAAGTCACCCG	TCAGTAGGGATACTTGAAGCC
**IsoB**	TGCTCTTCTGACCGGCGTTG	TCAGGTGCGCATACATACATG
**IsoC**	TGCTCTTCTGACCGGCGTTG	GAAACAAGTATCTAGGTTGG
**Isok**	GCCCGTGAGCCACTTATGC	TCAGTAGGGATACTTGAAG

### Polymerase chain reaction

Total RNA was extracted from undifferentiated and 8 days differentiated ESC using TRIzol Reagent (Invitrogen) following manufacturer's instruction. All samples were further treated with amplification grade DNase I (Sigma-Aldrich). Reverse transcription for cDNA synthesis was carried out on a thermal cycler using the RevertAid Reverse Transcriptase (Thermo Scientific Fermentas) to first strand synthesis system according to the manufacturer's protocol (Invitrogen). The transcription rates of selected mRNAs were measured by PCR using a thermal cycler. PCR was performed in 20 µl of buffer reaction containing of 2 µg cDNA, Maxima Hot Start Taq DNA Polymerase (Thermo Scientific Fermentas), and 5 pmol of each sequence-specific primers (Table 1). Thermal cycling conditions consisted of a denaturation for 10 min at 95°C followed by 40 cycles for denaturation for 15 s at 95°C, and annealing for 30 s at 60°C and extension for 1 min at 70°C, followed by final extension 10 min at 72°C. Ten microliters of the PCR reaction were analyzed on a 1.0% agarose gel containing ethidium bromide and visualized under ultraviolet light. The resulting bands were subjected to densitometric analysis with the Image J software. P2X7R isoform splicing variant expression levels were normalized by comparison to GAPDH gene expression.

### BrdU incorporation assay

Cell proliferation was measured following incubation with 20 µM 5-bromo-2-deoxyuridine (BrdU; Sigma-Aldrich) for 1 hour. The cells were fixed with ice-cold 75% ethanol for 10 minutes, washed with PBS, incubated for 30 min in 2.0 M HCl and neutralized with 0.1 M sodium tetraborate. The cells were blocked with a 2% fetal bovine serum, 0.1% Triton-X solution in PBS for 30 minutes. After washing with PBS, they were incubated for 1 h with rat anti-BrdU antibodies (Abcam; 1∶200 dilution). Alexa Fluor 488 secondary antibodies (Life Technologies) were used at 1∶1000 dilution. After another washing step, propidium iodide solution (Life Technologies) at 50 µg/ml was used as a DNA stain [28]. Cells, which had undergone through the whole process, but were not marked with the primary antibody and propidium iodide, were used as negative controls. The percentages of BrdU-positive cells and DNA contents were measured with the Attune flow cytometer (Life Technologies). Both fluorophores were excited by a 488 nm blue laser; Alexa Fluor 488 emission was captured by through a 530/30 nm filter and propidium iodide emission was captured through a 574/26 nm filter.

### Flow cytometry analysis

The cells were fixed with a 4% paraformaldehyde solution for 30 minutes, washed in PBS and blocked with a 2% fetal bovine serum, 0,1% triton-x solution in PBS for 30 minutes. The cells were then incubated in mouse anti-SSEA1 antibodies (Millipore, 1∶500 dilution) and either rabbit anti-Ki67 (Millipore, 1∶500 dilution) or rabbit anti-β3 tubulin antibodies (Sigma, 1∶700 dilution) for 1 hour. The cells were incubated for 45 min. with the Alexa Fluor 488 anti-rabbit and Alexa Fluor 647 anti-mouse secondary antibodies (Life Technologies, 1∶1000 dilution). Cells which had undergone through the whole process, but were not marked with the primary antibodies, were used as negative control. The percentage of marked cells was measured in the Attune flow cytometer (Life Technologies, CA). Alexa Fluor 488 was excited by a 488 nm blue laser and its emission was captured through a 530/30 filter. Alexa Fluor 647 was excited by a 638 nm red laser and its emission was captured through a 660/20 filter.

### Statistical Analysis

Comparisons between experimental data were made by one- or two-way analysis of variance following the Bonferroni post-test using GraphPad Prism 5.0 software (GraphPad Software Inc., San Diego, CA). Criteria for statistical significance were set at p<0.05 (*), p<0.01 (**), or p<0.001 (***).

## Results and Discussion

### Functional expression and subcellular localization of P2X7 receptors in undifferentiated and neural-differentiated mouse embryonic stem cells

**Figure 1:**
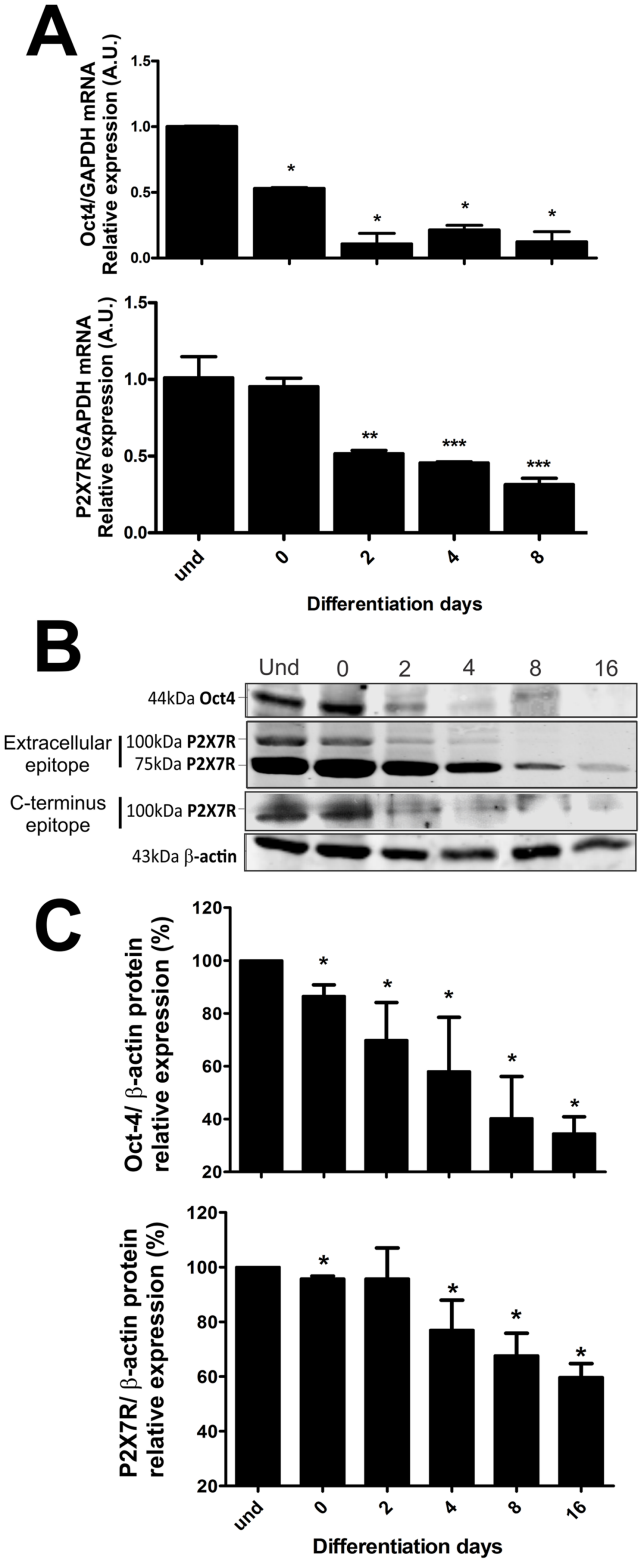
P2X7 receptor and Oct-4 expression in undifferentiated mouse ESC and cells induced to neural differentiation. P2X7 receptor and Oct-4 expression levels in undifferentiated (und) and cells induced to differentiation (days 0–8) were determined by (**A**) real-time PCR and (**B,C**) Western blotting assays as described in Materials and Methods. For real-time PCR, quantitative analysis of the relative expression of P2X7R and Oct-4 in E14Tg2A cell line was performed using GAPDH mRNA transcription rates as endogenous control for normalization of expression levels. For Western blotting, P2X7R and Oct-4 expression levels were obtained and analyzed by densimetric analysis of protein bands and were compared to β-actin expression levels. The P2X7R was identified by two antibodies, which recognize the extracellular or C-terminus domain. Bars represent mean ± standard errors (S.E.) of three independent experiments performed in triplicate. Data were analyzed for statistical relevance with the One-Way ANOVA test followed by the Bonferroni post hoc test (*p<0,05,**p<0,01, ***p<0.001 compared to control data).

ESC induced for 8–16 days with *all-trans* retinoic acid to neural differentiation [29] reveal neuron-specific morphology and expression patterns, such as β3-tubulin, and responsiveness to neurotransmitters and KCl, indicating expression of functional neurotransmitter receptors and voltage-operated ion channels [24]. In view of reports of purinergic signaling during early development [13], we correlate here Oct-4 and P2X7R gene and protein expression levels in pluripotent ESC and along neural differentiation of these cells (Figure 1A–C). P2X7R expression decayed during differentiation, such as it was observed for Oct-4. Down-regulation of Oct-4 has been reported when pluripotent cells undergo differentiation [30], [31].

In undifferentiated ESC, P2X7R expression pattern showed one band with a molecular weight of 75 KDa and another one with 100 KDa. During the progress of differentiation the expression of both isoforms decayed, but remained expressed (Figure 1B). On one hand, a molecular complex of approximately 100 KDa has been described as a result of a complex formed by P2X7 and P2X4 subunits [32], but this is not applicable, because we performed SDS-PAGE of total cell extracts. On the other hand, previously published experimental evidence suggests that the 75 KDa form could present the glycosylated and functional form of the P2X7 receptor [33]. In order to resolve this question, immunostaining with another polyclonal antibody that recognizes just the intracellular C-terminus domain, that is present only in the canonical isoform, revealed the 100 KDa species in ESC undergoing neural differentiation (Figure 1B). The immunostaining is reliable given that no 100 and 75 KDa band staining was detectable in brains of P2X7R (−/−) knockout mouse brains (Figure S1). Therefore, we suggested that detection of two different isoforms resulted from the expression of alternative splicing variants.

**Figure 2:**
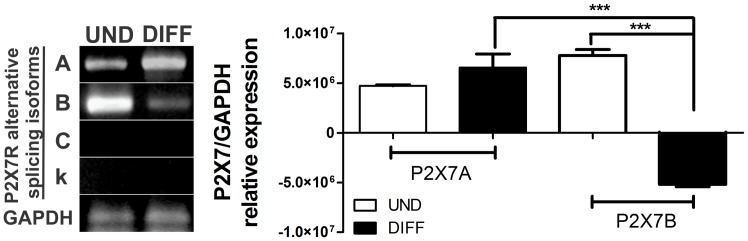
Differential expression of P2X7R alternative splicing isoforms in undifferentiated and neural- differentiated ESC. (**A**) P2X7 receptor isoform A, B, C and k expression in undifferentiated (UND) and cells following 8 days of neural differentiation (DIFF) was determined by RT-PCR. (**B**) P2X7R isoforms expression levels were obtained and analyzed by densimetric analysis of DNA bands and were compared to GAPDH expression levels. Data were analyzed for statistical relevance with the One-Way ANOVA test followed by the Bonferroni post hoc test (*p<0,05,**p<0,01, ***p<0.001 compared to control data).

In order to determine the expression pattern for the different P2X7 splice variants along the neuronal differentiation of E14Tg2a cells, RT-PCR with specific sets of primers (Table 1) for each variant was performed. As shown in Figure 2A, only the transcripts encoding the isoforms A and B were expressed both by undifferentiated and differentiated cells. It is noteworthy that the isoforms A and B correspond to the canonical full-length P2X7A variant and the truncated P2X7B variant, respectively. This truncated variant is identical with the P2X7A variant between residues 1–430, but it has a much shorter C-terminus (its C-terminus is just 87 amino acid residues long, in comparison to the P2X7A C-terminus with circa 240 amino acid residues). Although there is no statistical difference in the relative expression of the isoform A between undifferentiated and differentiated cells, the isoform A is more abundantly expressed than the isoform B in differentiated cells. On the other hand, the relative expression of the transcript encoding the isoform B decreased significantly when the cells differentiated (Figure 2B). Summing up, our results suggest that in undifferentiated cells both the P2X7 splice variants are equally expressed. But upon onset of differentiation, the relative expression of the variant A remains constant, while the relative expression of the variant B decayed significantly. These results are in agreement with the previous ones obtained in Western-blot experiments (Figure 1B). The two observed bands are likely to correspond to the P2X7A and P2X7B variants. This is reinforced by the fact that, by using an antibody against a C-terminus epitope, just the circa 100 KDa band is observed. This antibody is not capable of identifying the P2X7B variant, since this lacks the epitope. However, two bands are observed, when an antibody against the extracellular domain is used in Western-blot experiments (Figure 1B–C). It has been already demonstrated that the isoform B, different from the P2X7A variant, is not able to form a large pore, as indicated by bromide ethidium uptake analysis [34], [35]. We speculate therefore, that P2X7B isoform expression during the initial phase of differentiation may be a mechanism against cellular death allowing that more cells proliferate and differentiate, since pore formation leads eventually to apoptosis. In fact, it is possible that the P2X7B splice variant co-assembles with P2X7A receptors and suppresses cell permeabilization induced by the full-length P2X7A receptor. This mechanism has been already observed at least for the P2X7C variant [35].

**Figure 3:**
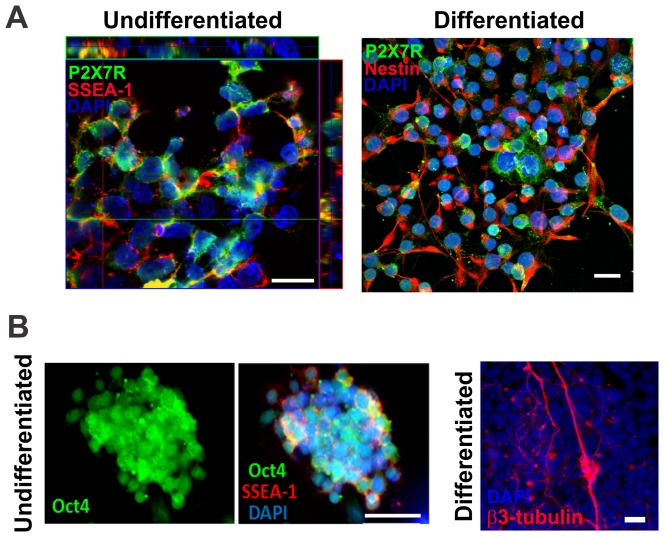
Immunofluorescence studies of P2X7 receptor and differentiation-stage specific proteins in undifferentiated and differentiated ESC. P2X7R expression and co-localization with differentiation-stage specific proteins was determined by confocal microscopy and immunofluorescence assays as described in Materials and Methods. (**A**). Left panel: Co-localization between SSEA-1 (stage-specific embryonic antigen-1) and P2X7R immunofluorescence in undifferentiated ES cells. Co-localization of the protein is shown by Z-stack analysis. Right panel: Double-immunostaining for nestin (neural stem and precursor marker) and P2X7R expression in cells induced to differentiation for 8 days. (**B**). Left panel: Immunostaining for Oct-4 (pluripotency marker) in undifferentiated cells. Middle panel: Immunostaining for Oct-4 and SSEA-1 in undifferentiated cells. Right panel: Staining pattern for the neuron-specific marker β-tubulin in neural-differentiated cells. Cell nuclei were visualized by DAPI staining. Scale bar, 50 µm.

Undifferentiated ESC express P2X7R altogether with SSEA-1, which is a membrane carbohydrate typically found in stem cells [36], as shown in Figure 3. The overlay of immunofluorescence images revealed co-expression as yellow regions, suggesting that the P2X7R is functionally expressed at the plasma membrane. In neural-differentiated cells (day 8) with diverse morphologies, neurons (β3-tubulin positive cells) and neural precursors (nestin-positive cells) expressed the P2X7R throughout the cell (Figure 3).

**Figure 4:**
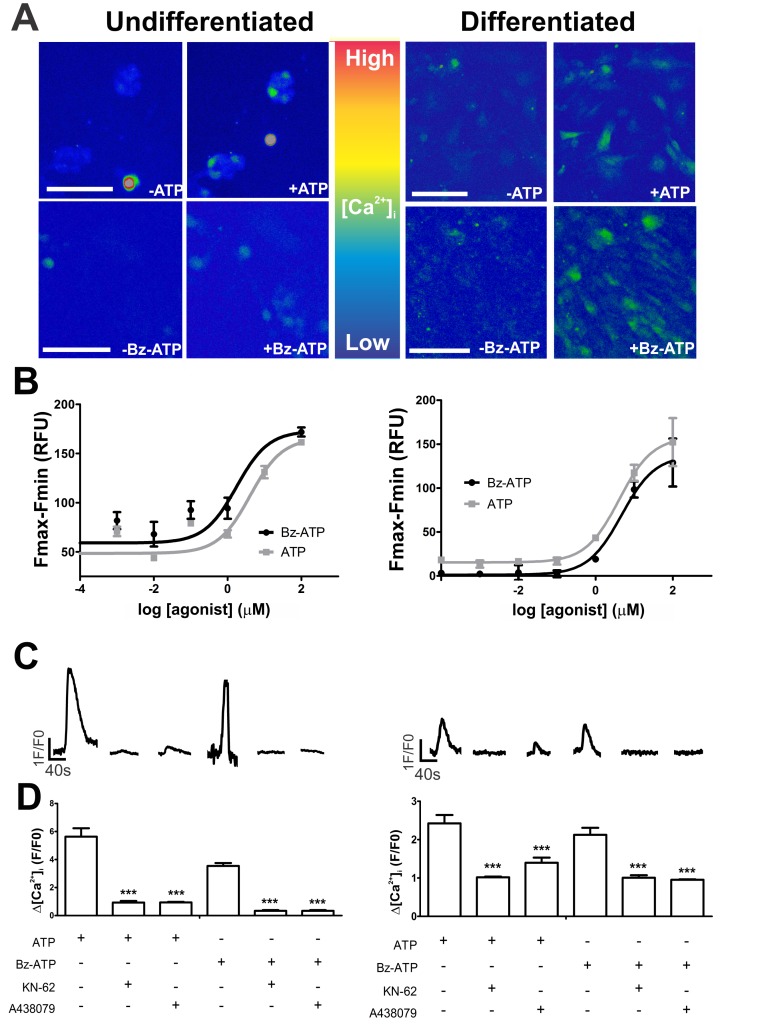
ATP- and Bz-ATP-induced intracellular calcium fluxes in undifferentiated and neural-differentiated ESC. ATP- and Bz-ATP-evoked P2X7R responses were determined by single-cell calcium imaging and microfluorimetry assays as described in Materials and Methods. (**A**) For calcium imaging, 10 µM of purinergic receptor agonists, ATP or Bz-ATP, were used to stimulate receptor-mediated intracellular calcium fluxes in undifferentiated and neural-differentiated cells (day 8). (**B**) For microfluorimetry-based calcium measurements, increasing concentrations of ATP or Bz-ATP were added and time kinetics of changes in fluorescence emission was recorded. Peak values of measured [Ca^2+^]_i_ transients are reported. Nonlinear Regression was used for curve-fitting and calculating the EC50 values. (**C and D**) P2X7R inhibitors were studied regarding their effects in blocking ATP- and Bz-ATP-induced [Ca^2+^]_i_ transients, as studied by calcium imaging. For these experiments, cells were pre-treated for 2 min with 10 µM KN-62 or 1 µM A438079 and then stimulated with 10 µM ATP or Bz-ATP. Data represent mean values ±S.E. of three independent experiments performed in triplicate.

In agreement with functional expression of purinergic receptors, we analyzed the induction of intracellular calcium transients ([Ca^2+^]_i_) in undifferentiated and neural-differentiated cells by the purinergic agonists ATP and Bz-ATP, which is a selective P2X7R agonist [37], [38], [39], by calcium imaging and microfluorimetry (Figure 4). Dose-response curves revealed EC_50_ values of 4.1±1.8 µM and 1.7±2,2 µM in undifferentiated cells and 4.1±1.4 µM and 4,7±1.5 µM in neural-differentiated cells for stimulation by ATP and Bz-ATP, respectively (Figure 4A–B). ATP (10 µM) and Bz-ATP (10 µM) activated P2X7R function in both undifferentiated and neural-differentiated cells, as agonist-stimulated transients were abolished following pretreatment for 2 min with the P2X7R inhibitors KN-62 (10 µM) [39], [40], [41] and A438079 (1 µM) [42] (Figure 4C–D).

Neural-differentiated cells revealed EC_50_ values for ATP and Bz-ATP that are not statistically different from those observed in undifferentiated cells (Figure 4B). However, agonist concentrations needed for activating this receptor were higher and response amplitudes decreased in neural-differentiated cells, suggesting that differentiated cells express P2X7R forms that are slightly less sensible to agonists than those in undifferentiated ESC. Differences in calcium responses in undifferentiated and differentiated cells may be related to expression levels of 100 and 75 KDa forms of the receptor (isoforms A and B) with the 100 KDa isoform A possibly being more sensible to activation by Bz-ATP than the 75 KDa isoform B.

Many studies have shown that activation of P2X7R needs higher concentration of agonists (100–1000 µM of ATP) [43], usually resulting in cell death; however, Francesco Di Virgilio showed recently that this receptor can be activated by lower concentrations, like we found, and induces trophic effects, mainly in tumor cells [44]. Therefore, in agreement with the here shown dose-response curves, low P2X7R agonist concentration would maintain undifferentiated stem cell functions, including pluripotency and proliferation, while higher agonist concentration, necessary for P2X7R activation in differentiated cells, would lead to different, possibly undesired effects. Such hypothesis is in agreement with the here observed P2X7R expression down-regulation following induction of ESC to neural differentiation.

Expression of P2X7R in neural-committed cells has been documented previously. For instance, embryonic rats (E15.5) express P2X7R mRNA together with the neural stem and progenitor cell marker nestin [45]. Thus, P2X7R expression levels also depend on the differentiation stage in other cellular models. Neuro-2a cells, derived from spontaneous mouse neuroblastoma, respond to retinoic acid-induction with the onset of neural differentiation together with a decrease in the expression and activity of P2X7 receptors [46]. In line with these results, RA-treated SH-SY5Y cells exhibited a neuron-like phenotype with neurites extending more than twice the length of the cell body and cell growth arrest simultaneously with down-regulation of P2X7R expression. The here cited example corroborate our hypothesis that differential expression and activity patterns of P2X7R are necessary for guiding ESC differentiation into neural phenotypes [47].

### Promotion of cell cycle entry by P2X7 receptor activity

ES cells are derived without the intervention of any immortalizing agent, do not undergo senescence, they proliferate without apparent limit and are not subject to contact inhibition or anchorage dependence. In fact, no means of inducing cell-cycle arrest and quiescence in ES cells are known [48].

As already cited above, Di Virgilio and co-workers found that P2X7R promoted proliferation in tumor and microglial cells when stimulated by agonist [44]. Lemoli et al. showed that ATP modulates human hematopoietic stem cell proliferation by acting as potent early growth factor *in vitro* [49]. Thus microglial cell proliferation is blocked when P2X7R is inhibited [50]. In agreement, tumors are induced to proliferation upon P2X7R activation [51].

**Figure 5:**
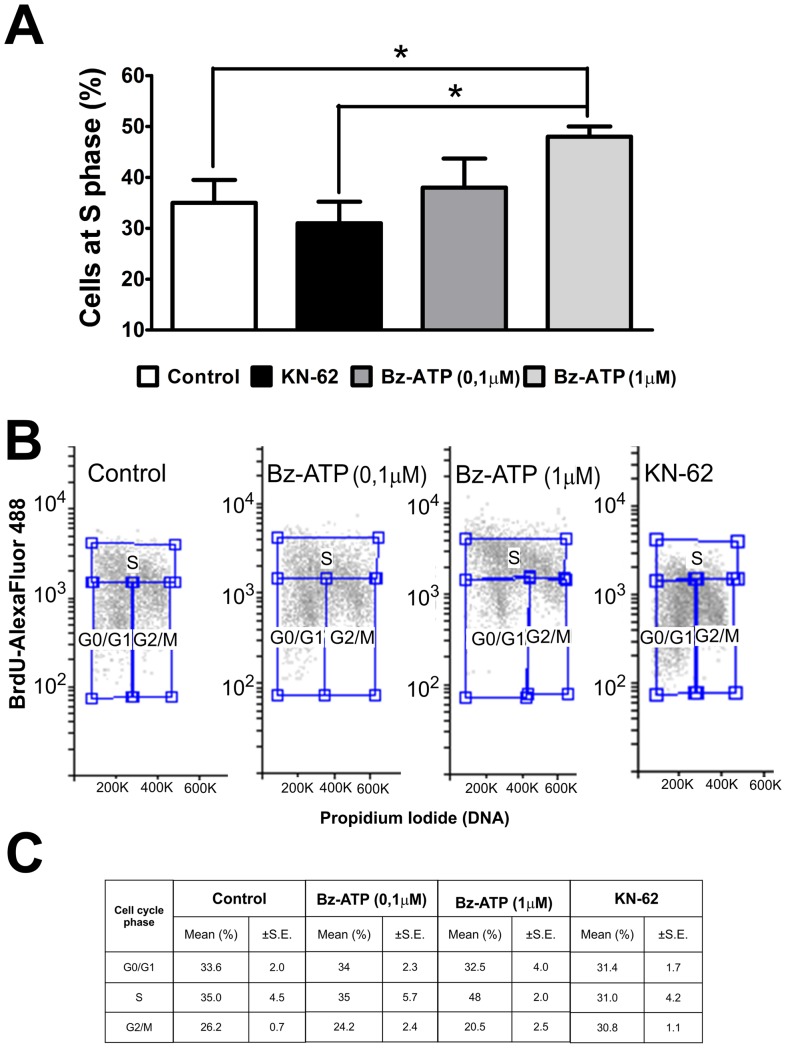
Proliferation of ESC in conditions of P2X7R modulation. Cell cycle analysis based on flow cytometric analysis of BrdU incorporation and propidium iodide DNA-staining were performed as described in Materials and Methods. (**A**) Cell distributions at different S-phases of ESC treated with P2X7R agonist or inhibitor, 0.1 µM and 1 µM Bz-ATP and 10 µM KN-62 for 96 h, respectively. Shown data are representative for mean values ±S.E. of five independent experiments. Data were statistical analyzed by the One-Way ANOVA test followed by the Bonferroni post hoc test with (*p<0,05 compared to control data). (**B**) Representative BrdU/PI cell cycle analysis of ESC treated with Bz-ATP or KN-62 compared to untreated control cultures. (**C**). Cell cycle distributions of ESC treated for 96 h with the P2X7R agonist (0.1 µM or 1 µM Bz-ATP) or the P2X7R inhibitor KN-62 (10 µM), respectively. Shown data are representative for mean values ±S.E. of five independent experiments.

**Figure 6:**
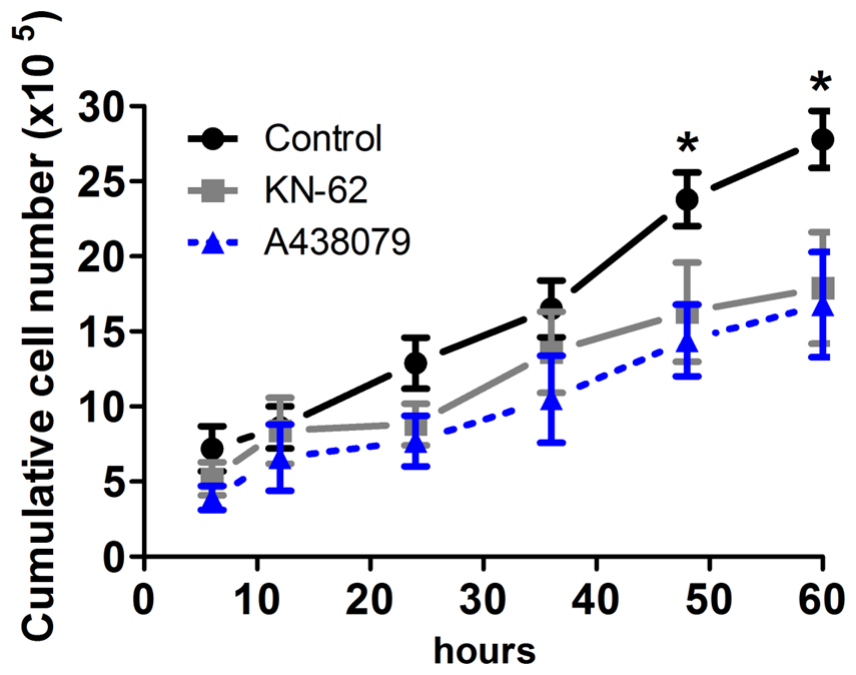
Inhibition of embryonic stem cell growth in the presence of P2X7R inhibitors. Cells were plated at 3X10^5^ cell/ml density and P2X7R was inhibited daily with 10 µM KN-62 or 1 µM A438079 and then the number of cells were counted. Data represent mean values ±S.E. of three independent experiments performed in triplicate (*p<0,05 compared to control).

In order to understand modulation of ESC proliferation by P2X7R, we analyzed cell cycle distributions and cell cycle entry rates of undifferentiated cells cultured for 96 h in the absence or presence of Bz-ATP (0.1 and 1 µM) or the selective P2X7R inhibitor KN-62 (10 µM) [41]. Here, we provide evidence for an increase in the percentage of cells in S-phase (from 35% to 48%) in conditions of 1 µM Bz-ATP treatment. Accordingly, cells exposed to KN-62 revealed a decrease in the percentage of cells in S phase (from 48% to 31%) (Figures 5A–C). Corroborating these data, cell growth curve assays showed a delay in proliferation of ESC treated with the P2X7R inhibitors KN-62 and A438079 (Figure 6). These results indicate that P2X7R activity results in ESC proliferation by accelerating entry into the cell cycle.

A possible underlying mechanism could be that the P2X7R contributes to cell cycle-dependent [Ca^2+^]_i_ transients, which are required for G1/S progression of mouse embryonic stem cells [52]. ESC usually overpass the G1 checkpoint to proliferate faster having a short G1 phase of 1.5 h [53], [54]. G1 phase duration is extended in order to induce ESC differentiation commitment [55], [56]. In view of that, in our work P2X7R modulation did not change the percentage of cells in G1 phase (Figure 5B–C), possibly indicating that the P2X7R is involved in proliferation, without affecting pluripotency, since P2X7R activity modulation did not affect Oct-4 marker expression (Figure S2).

### P2X7 receptor modulates mouse neural precursor differentiation

The importance of P2X7R on ESC survival at the undifferentiated state has been reported previously [5]. Moreover, previous results obtained by our laboratory using P19 embryonal carcinoma cells stably expressing shRNA for degradation of P2X7R coding mRNA established the importance of the P2X7R for glial cell proliferation and differentiation and the ratio of glial over neural cells following induction with retinoic acid [57]. Here, using ESC pre-differentiated to neural precursor cells, we show the importance of P2X7R activity modulation for the onset of ESC neuroectodermal differentiation and neuroblast maturation beyond the results obtained with P19 EC cells.

**Figure 7:**
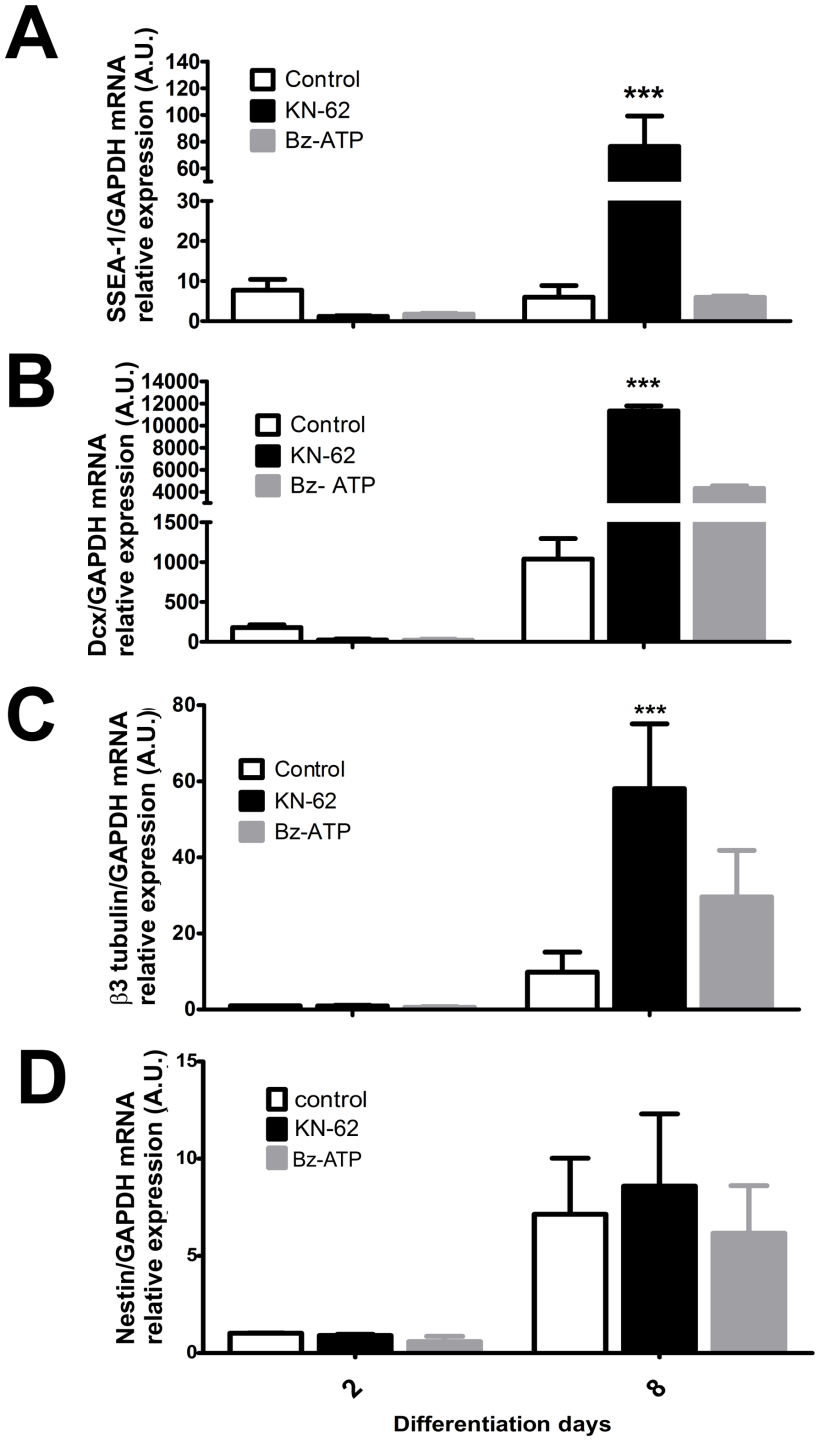
Effects of P2X7R agonists and antagonists on the progress of neural differentiation, studied on the gene expression level. Total RNA was isolated from cell cultures, and real-time PCR reactions were performed as described in Materials and Methods. Quantitative analysis of the relative expression of (**A**) SSEA-1, (**B**) Dcx, (**C**) β3-tubulin and (**D**) nestin in E14Tg2A cell line were performed by real time-PCR where GAPDH expression was used as internal control for normalization of expression levels. ESC were treated with P2X7R agonists or inhibitors (10 µM Bz-ATP or 10 µM KN-62) respectively. Data were analyzed by the Two-Way ANOVA test followed by the Bonferroni post-hoc test. The experiments were performed three times in triplicate with (***p<0.001 compared to control).

For this purpose, cells were induced to neural differentiation in the absence or presence of Bz-ATP (10 µM) or KN-62 (10 µM) along differentiation followed by gene expression analysis of the following differentiation stage markers: SSEA-1, expressed by stem cells undergoing neuroectoermal differentiation [58], [59]; nestin, doublecortin (Dcx), expressed by migrating and differentiating neurons [60]; and β3-tubulin, characterizing young neurons. The presence of KN-62 led to an increase of SSEA-1 (>8,5 fold), Dcx (>9,1 fold) and β3-tubulin (>4,8 fold) gene expression (Figure 7A–C), while nestin (Figure 7D) and GFAP (glial marker, data not shown) expression levels were not affected. These results suggest that P2X7 receptor blockade promotes neurogenesis, but not gliogenesis, being in agreement with our previous study [57].

**Figure 8:**
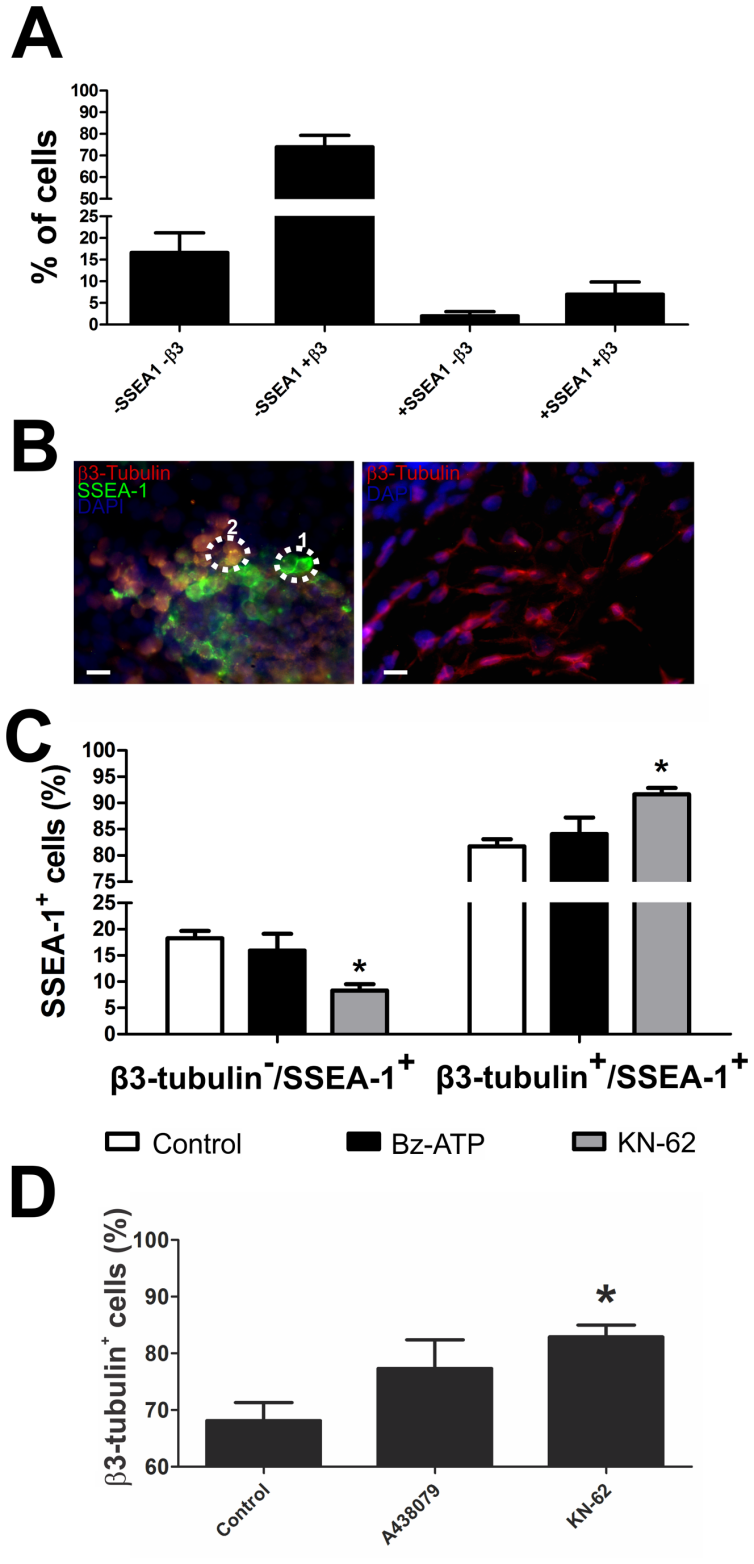
Modulation of neuroectodermal differentiation of mouse ESC by P2X7R activity. (**A**) Flow cytometry analysis of SSEA-1 and β3-tubulin protein expression in neural-differentiated ES cells. Cells were cultured and induced to differentiation as described in Materials and Methods. (**B**) Immunofluorescence assay of SSEA-1 and β3-tubulin expression of cells following 8 days of differentiation. Circled areas: **1**. cells expressing only SSEA-1, **2**. cells co-expressing SSEA-1 and β3-tubulin. Scale bar, 50 µm. (**C**) Flow cytometry analysis of SSEA-1-positive cells co-expressing or not β3-tubulin on day 8 of differentiation cultured in the presence of 10 µM Bz-ATP or 10 µM KN-62, respectively. (**D**) Percentage of β3-tubulin-positive cells differentiated in the absence or presence of 1 µM A438079 or 10 µM KN-62, as determined by flow cytometry. Statistical relevance was analyzed by the One-Way ANOVA test followed by the Bonferroni post-hoc test. Six independent experiments were performed (* p<0.05 compared to control data).

We observed by flow cytometry and by immunofluorescence assays that the population of differentiating cells is heterogeneous with around 70% of cells expressing only β3-tubulin and revealing neuronal morphology and neurite extensions (Figure 8A and B). A population of 15% was negative for expression of SSEA-1 and β3-tubulin, and around 15% of cells were SSEA-1 positive within 80% of them co-expressing β3-tubulin. These β3-tubulin-/SSEA-1-positive cells revealed morphological characteristics different from neurons; they are sphere shaped similarly to supposedly undifferentiated cells expressing SSEA-1, but not β3-tubulin (Figure 8 A and B).

P2X7R inhibition promoted neuronal differentiation, as the presence of KN-62 led to an increase of the number of β3-tubulin-positive cells within the SSEA-1 positive cells and decreased the β3-tubulin negative cells (Figure 8C). Probably, the SSEA-1^+^/β3-tubulin^−^ cells are less differentiated than SSEA-1^+^/β3-tubulin^+^, and the inhibition of P2X7R led the neuronal commitment of SSEA-1^+^/β3-tubulin^−^ cells.

SSEA-1 is a carbohydrate adhesion molecule [61] expressed in various types of stem cells, including the adult brain where neural stem cells reside in specific niches [58], [59], [62]. SSEA-1 is strongly expressed by neuroepithelial cells, being downregulated upon further differentiation, which suggests that delamination from the rosette-like structures parallels surface expression changes of these glycolipid markers [63], [64]. This is consistent with the observation that SSEA-1 is present on neural stem cells *in vivo*, residing in stem cell niches of the adult brain [58]. Moreover, β3-tubulin can be expressed in immature neurons in the proliferative ventricular and subventricular zones of the developing telencephalon distinguishing two neuronal populations: those that remain for an indefinite period of time in the proliferative zones, and those that leave the proliferative zones soon after being generated and migrate independently from radial glial fibers [65]. In early development, dividing neuronal-committed precursors express β3-tubulin [66]. Such kind of precursor present in developing brain is the neuroblast, a cell that divides and can differentiate into a neuron after a migration phase [67]. These cells also express β3-tubulin and are originated by neural stem cell differentiation, mainly from neuroephitelial cells expressing SSEA-1, and are committed to the neuronal fate.

**Figure 9:**
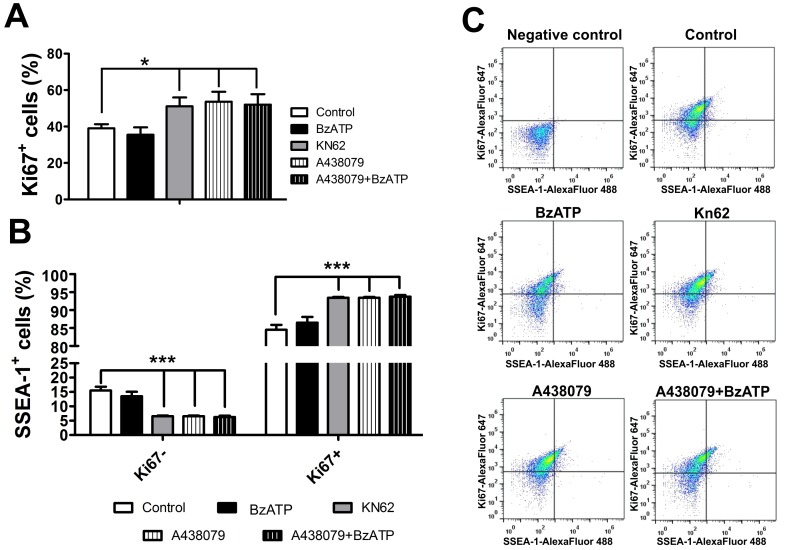
Expression of the proliferation antigen Ki-67 in SSEA-1 positive cells differentiated in the absence or presence of P2X7R agonists and antagonists. (**A**) Flow cytometry determination of Ki67^+^ cells following neural differentiation for 8 days in the absence or presence of the agonist Bz-ATP (10 µM), the antagonists KN-62 (10 µM) or A438079 (1 µM), or Bz-ATP and A438079. (**B**) Determination of percentages of Ki67^−^/SSEA-1^+^ and Ki67^+^/SSEA-1^+^ cells for experimental conditions explained in A. (**C**) Representative dot-plot images for Ki67/SSEA-1 double staining for conditions described in B. Statistical relevance was analyzed by the One-Way ANOVA test followed by the Bonferroni post hoc test. Bars represent mean ± SE of 5 independent experiments (*p<0,05, ***p<0.001 compared to control data).

Co-expression analysis of SSEA-1 and Ki-67, a marker of proliferating cells, by flow cytometry revealed that a few cells were SSEA-1^+^/Ki67^−^, while the larger percentage of the population represents proliferating neuroblasts in agreement with their characteristics as dividing cells [68] (Figure 9A). Within the population of SSEA-1 positive cells, blockade of P2X7R activity by KN-62 or A438079 led to an increase in the number of Ki67^+^ cells (Figure 9B and C), recruiting progenitors into the cell cycle and differentiation. Taken together, SSEA-1^+^/β3-tubulin^−^ cells may be stem cells (neuroephitelial) giving rise to neuroblasts (SSEA-1^+^/β3-tubulin^+^) that finally differentiate into neurons (SSEA-1^−^/β3-tubulin^+^). P2X7R should then be less expressed to favor neuroblast differentiation.

## Conclusions

We have provided experimental evidence for novel functions of the P2X7R in ESC biology: P2X7R expression and activity is upregulated in embryonic cells, maintaining ESC proliferation, while upon induction to neural differentiation P2X7 receptor expression and activity needs to be suppressed. Pharmacological inhibition of P2X7R activity results in a higher percentage of ESC undergoing neuroectodermal differentiation, in the rescue of quiescent progenitors into cell cycle and in the promotion of differentiation into neurons. Our data suggest that extracellular nucleotides may provide a novel and powerful tool for modulating ESC functions.

## Supporting Information

Figure S1 **Western blot with anti- P2X7R antibody in brain extracts of P2X7R (−/−) knock-out animals.** P2X7 receptor expression was determined by Western blotting assay as described in Materials and Methods. For Western blotting, lysates of P2X7−/− knockout animal brain and undifferentiated ESC were used to measure expression of P2X7R with the antibody that recognizes the extracellular domain of the receptor.
(TIF)

Figure S2 **Oct-4 expression during neural differentiation of ESC in conditions of P2X7R inhibition.** Oct-4 expression was determined by real-time PCR assay as described in Materials and Methods. Prior to real-time PCR, cells were induced to differentiation in the absence or presence of 1 µM Bz-ATP or 1 µM KN-62. Relative expression levels of Oct-4 in E14Tg2A cell line were calculated using GAPDH mRNA transcription rates as endogenous control for normalization of expression levels. Bars represent mean ± standard errors (S.E.) of three independent experiments.
(TIF)
