# Supplementary figures and images for: Modulation of Mouse Embryonic Stem Cell Proliferation and Neural Differentiation by the P2X7 Receptor
Source: PLoS One. 2014 May 5;9(5):e96281. doi: 10.1371/journal.pone.0096281 (PMC4010452; doi:10.1371/journal.pone.0096281)

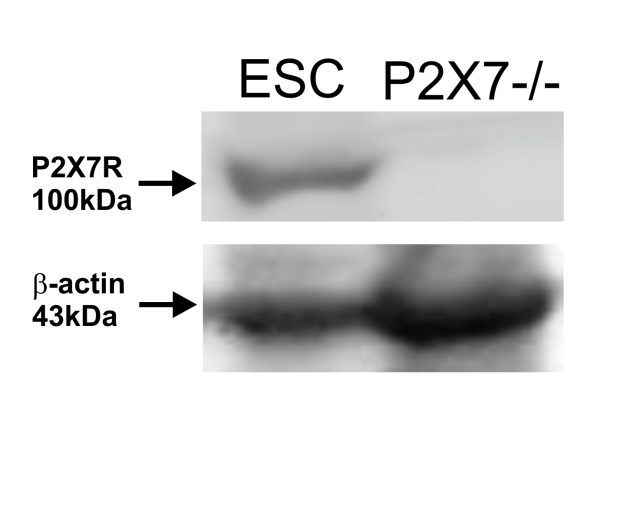

Supplement: Figure S1 — Western blot with anti- P2X7R antibody in brain extracts of P2X7R (−/−) knock-out animals. P2X7 receptor expression was determined by Western blotting assay as described in Materials and Methods. For Western blotting, lysates of P2X7−/− knockout animal brain and undifferentiated ESC were used to measure expression of P2X7R with the antibody that recognizes the extracellular domain of the receptor. (TIF) [file pone.0096281.s001.tif]

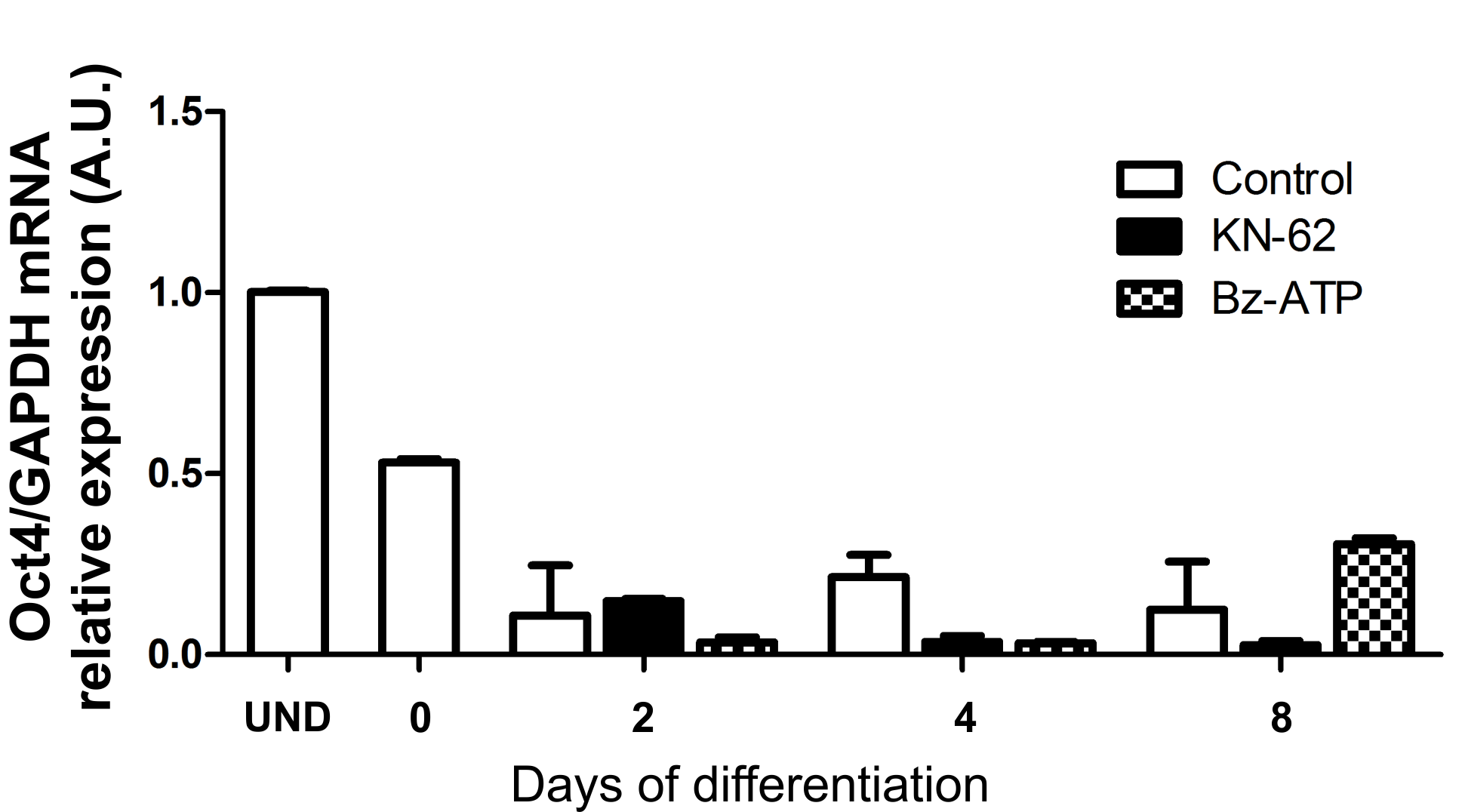

Supplement: Figure S2 — Oct-4 expression during neural differentiation of ESC in conditions of P2X7R inhibition. Oct-4 expression was determined by real-time PCR assay as described in Materials and Methods. Prior to real-time PCR, cells were induced to differentiation in the absence or presence of 1 µM Bz-ATP or 1 µM KN-62. Relative expression levels of Oct-4 in E14Tg2A cell line were calculated using GAPDH mRNA transcription rates as endogenous control for normalization of expression levels. Bars represent mean ± standard errors (S.E.) of three independent experiments. (TIF) [file pone.0096281.s002.tif]
